# Social measures for reducing exposure to secondhand smoke in migrant workers of sugarcane harvest in the lower northern region of Thailand

**DOI:** 10.18332/tid/140138

**Published:** 2021-09-23

**Authors:** Narongsak Noosorn, Anukool Manoton, Rishad Choudhury Robin

**Affiliations:** 1 Faculty of Public Health, Naresuan University, Phitsanulok, Thailand; 2 School of Medicine, University of Phayao, Phayao, Thailand; 3 Ministry of Health and Family Welfare Coordination Centre, Cox’s Bazar, Bangladesh

**Keywords:** Thailand, secondhand smoke, social measures, migrant worker, health protection

## Abstract

**INTRODUCTION:**

The sugarcane harvest migrant workers are an underprivileged group in Thailand and have a high risk of exposure to secondhand smoke but are potentially neglected in health promotion interventions.

**METHODS:**

This three-phase study applied a mixed-method research approach. The data were collected from February to December 2019 from the Sukhothai province of Thailand. In Phase 1, the level of secondhand smoke exposure of the sugarcane harvest migrant workers at the worker camp was explored. The data were collected from 462 workers by questionnaires and from 24 sample participants in the group discussions about the factors leading to the exposure to secondhand smoke. Phase 2 was the provision and implementation of social measures for the health protection of migrant workers and families from exposure to secondhand smoke. In Phase 3, an evaluation of the health protection model for the migrant workers and families from secondhand smoke exposure was explored.

**RESULTS:**

Workers aged ≤40 years had 1.9 times higher exposure to secondhand smoke than workers aged ≥41 years (OR=1.93; 95% CI: 1.24–3.01). Those who worked overtime had 1.7 times higher exposure to secondhand smoke than those who did not work overtime (OR=1.71; 95% CI: 1.10–2.66). Social measures to prevent secondhand smoke were: given a warning, no rewards for cigarettes, designated smoking area, not asking the children to buy cigarettes, stop displaying cigarettes at grocery shops, and empowering woman to go against the smoking husband in the camp and the sugarcane field when the women, children, and nonsmokers are present. After implementing the measures, there was no exposure to secondhand smoke inside the room, cooking area, and at the quad in the camp center.

**CONCLUSIONS:**

Appropriate social measures for health protection can help to reduce exposure to secondhand smoke.

## INTRODUCTION

Tobacco smoking is one of the top public health issues, and about 1.1 billion people smoke different forms of tobacco around the world^1,2^. Around 7 million people die from a diverse use of tobacco, and for low- and middle-income countries, it is a tremendous burden because approximately 80% of the world’s smokers live in this part of the world^2^. Besides, male smoker prevalence is high in the Western Pacific region, and for female smokers, Europe has the highest prevalence^3^.

Globally, secondhand smoke (SHS) exposure is also an enormous burden as SHS is also responsible for 1.2 million deaths^4^. SHS exposure has no safe limit^2^. In developed countries, smoking causes 90% of lung cancers in men and up to 86% in women^4,5^. In the case of SHS exposure, the health effects are also huge as there are more than 7000 chemicals found in tobacco smoke, and many are responsible for cancer. Consequently, in adults, it causes different cardiovascular diseases such as heart attack, stroke, and other kinds of cancer^6^.

According to the Global Adult Tobacco Survey (GATS) by World Health Organization (WHO), the prevalence of tobacco smoking in Thailand is 23.7%, whereas, among males, it is 45.6% and 3.1% among females. The GATS data show that the overall SHS exposure prevalence inside the home is 39.1% and 27.2% in the workplace^7^. Smoking is common among laborers^8^. However, controlling smoking in the workplace pointedly reduces SHS exposure^9^. Moreover, incentives may be cost-effective in increasing quitting smoking in the workplace setting^10^.

Sukhothai is one of the major provinces of sugar cane planting in Thailand, and several sugar factories employ hundreds of seasonal informal sugarcane harvest workers each year^11^. According to the legal provision regarding informal labor and the Non-Smokers’ Health Protection Act, B.E. 2535, the sugarcane harvest migrant workers are considered underprivileged. They are temporarily employed and do not have employment contracts or specific rates for wages or compensation. They have a high risk of exposure to secondhand smoke but are neglected for health protection^12^. For this reason, this study was conducted to ascertain exposure to secondhand smoke by the informal sugarcane harvest migrant workers at the worker camp and to implement the social measures through a pre- and post-implementation assessment for the health protection of the informal sugarcane harvest migrant workers from SHS exposure.

## METHODS

**Figure 1:**
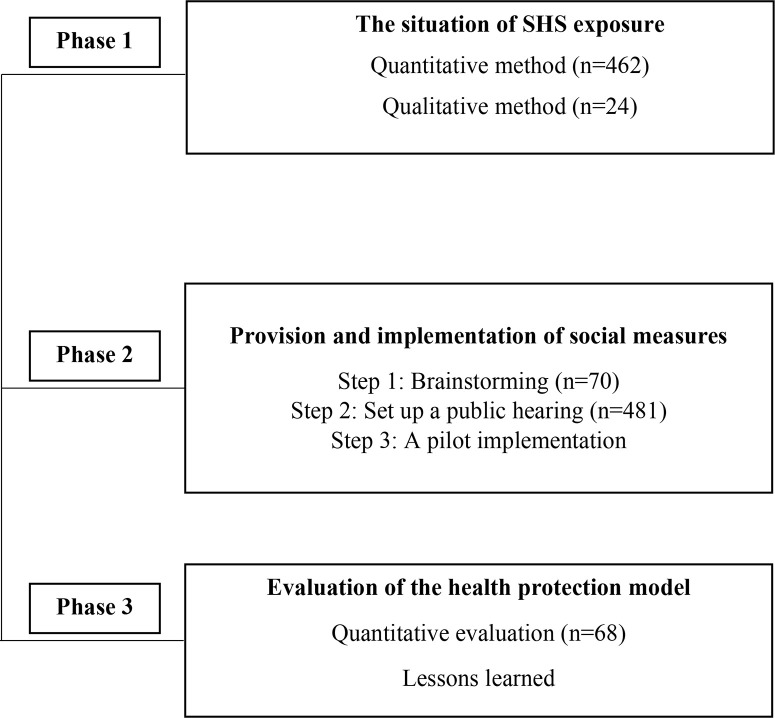
Study method

This study applied a mixed-method research approach and was divided into three phases (Figure 1). The data were collected from February to December of 2019.

### Phase 1: The situation of secondhand smoke exposure of the sugarcane harvest migrant workers at the worker camp

#### Quantitative method

The study area was selected by the purposive method from the provinces with the highest number of sugarcane harvest migrant workers in the lower northern region. Sukhothai was chosen because it was the primary source of sugarcane harvesting in Thailand. The number of informal sugarcane harvest workers was higher than for other provinces in the lower northern region. By using the Cochran formula, 462 migrant workers had been randomly selected^13^. A questionnaire was used as a tool divided into general information involving gender, age, marital status, education level, income, duration staying in the worker camp, overtime work, and the extent of SHS exposure. Collected data were analyzed using SPSS version 20 for Windows (IBM Corp., Armonk, NY). Descriptive statistics were used to describe basic sociodemographic features, whereas differences between categorical variables were assessed for significance using the chi-squared test or Fisher’s exact test, as appropriate. Binary logistic regression models were used to investigate the association between independent and dependent variables.

#### Qualitative method

A total of 24 sample participants comprised five smokers, three employers, four wives of smoker husbands, three heads of the workers, two cigarette shop keepers, two local government officers, two public health officers, and three non-smoking workers. Group discussion on the smoking behavior and factors leading to SHS exposure was employed. The focus group discussion consisted of questions about the general context of sugar cane harvest workers, worker camp lifestyle, the environment in camp, number of workers, the background of worker, smoking behavior of workers, the reason for smoking, mode of a new smoker, the context of SHS, and factors leading to secondhand smoke exposure. Data were collected from two discussion groups with a total of 12 members in each group. The discussion duration was 90 minutes. The data were analyzed with transcription and qualitative content analysis to categorize the items based on the research objectives^14^.

### Phase 2: Provision and implementation of social measures for health protection of the migrant workers and families from SHS exposure

Process of social measures provision and implementation comprised the three following steps.

#### Step 1

A total of 70 representatives included sugarcane harvest migrant workers and families, employees, worker leaders, shop keepers, local government officials, public health officers, and the researchers were selected purposively for the brainstorming session. The result of Phase 1 was used for the topic of the brainstorming session and the draft of the social measures was determined in line with the consensus among the stakeholders.

#### Step 2

We set up a public hearing with 462 workers from 10 worker camps, 10 employers, six shop owners, and three public health officers. The public hearing process referred to people’s opinion towards SHS problems and their solution through general public discussion, moderated by researchers and other government agencies. A public hearing gives the people’s opinion, and their feedback consisted of the social measures for the health protection of the informal sugarcane harvest migrant workers from SHS exposure. Lastly, we summarized the public’s comments and modified social standards and announced the final social measures.

#### Step 3

A pilot implementation of the social measures was conducted at four worker camps selected by the volunteer sampling method. The measures were: give a warning, not giving rewards by cigarettes, designate a smoking area, not smoking in the toilet, not convincing others to smoke, not asking the children to buy cigarettes, not dropping the cigarette butts where children may see, reducing the smoking frequency, a reminder from the family members, recommendations by ex-smokers, not displaying cigarettes and giving credit to buy cigarettes in the grocery stops, monitor the imitation of children, set up a positive environment for health, women empowerment, minimize free time to be distracted by smoking, not keeping cigarettes in a visible place at the neighboring camp and not smoking near the main hall where women and children are watching television (Table 1).

**Table 1:** Social measures to prevent secondhand smoke exposure in migrant worker camps

*Social measures*	*Methods*
1. Give a warning	Assign the employer and head of workers to give a warning. Set the rule to encourage the workers to respect each other. Give cooperation to accept the warning and surveillance.
2. No rewards by cigarettes	Set the rule to prohibit the employer from rewarding the workers with cigarettes.
3. Designate a smoking area	The employer, head of workers, and smokers designate the smoking area in the camp and announce it to the workers.
4. Do not smoke in the bedroom and toilet	The rule of the smoking ban in the bedroom and toilet.
5. Do not convince others to smoke	Smokers should not convince non-smokers to try smoking.
6. Do not ask the children to buy cigarettes	The children should not be asked to buy cigarettes.
7. Do not drop cigarette butts where children can see	Smokers should not drop cigarette butts in the camp area to prevent smoking imitation by the children.
8. Reduce the smoking frequency	Convince smokers to minimize smoking.
9. A reminder from the family members	A reminder and warning within the family.
10. Ex-smokers give recommendations to others	Assign the ex-smokers to give advice.
11. The grocery shops stop displaying cigarettes and giving credit to buy them	Ask for cooperation from the cigarette shop not to display the cigarettes or offer credit.
12. Monitor the imitation of children	The parents monitor imitation behavior in children.
13. Set a positive environment for health	Aim for a smoking-free camp. Display no-smoking signs around the camp. Arrange smoking areas outside the camp.
14. Women are empowered to go against the smoking husband in the camp and the sugarcane field when the women, children, and non-smokers are present	Support females in the camp to resist and prohibit the husbands from smoking in the camp and sugarcane field when the women are present.
15. Minimize free time to be distracted by smoking	Arrange activities during free time to distract from smoking, such as fishingnet weaving, wickerwork.
16. Do not put cigarettes in a visible place at the adjacent camp	Do not place cigarettes in a visible place where people can easily take them at the adjacent camp, walkway, toilet, and the main hall.
17. Do not smoke near the main hall where women and children are watching television	The main hall in the camp center is where the workers gather to chat and watch television. Do not smoke near the hall where women and children are watching television.
18. Reduce the frequency of drinking in the camp to minimize smoking while drinking	Reduce the frequency of drinking and smoking in the camp.

### Phase 3: Evaluation of the health protection model for the migrant workers and families from secondhand exposure

#### Quantitative evaluation

The survey form about the exposure location of secondhand smoke was used. Data of the first part were collected from the four trial camps. The trial results were evaluated after one month of implementation compared to the number of workers exposed to secondhand smoke.

#### Lessons learned and participatory evaluation

The researchers and workers gave their opinion systematically to monitor and evaluate the factors of success. Data were collected from all research participants. The researchers and workers collaborated to analyze and summarize the evaluation results.

## RESULTS

### Quantitative method

The results showed that 71.2% of the workers were male while 28.8% were female, and 58.7% were aged ≥41 years. A total of 40.5% were married, while 62.3% graduated from primary school. A total of 66.0% stayed in the worker camp for more than two years, and 74.0% reported a cigarette shop at their worker camp (Table 2). Besides, it was found that 64.7% were exposed to secondhand smoke, whereas 35.3% were not. Table 3 shows that age, marital status, and occupation were related to secondhand smoke exposure with statistical significance (χ^2^=14.69, p<0.001), (χ^2^=17.32, p<0.001), and (χ^2^=4.75, p=0.031), respectively. From Table 4, it can be seen that workers aged ≤40 years had 1.93 times higher exposure to secondhand smoke than those aged ≥41 years (OR=1.93; 95% CI: 1.24–3.01). Moreover, those who worked overtime had 1.71 times more exposure to secondhand smoke than those who did not work overtime (OR=1.71; 95% CI: 1.10–2.66).

**Table 2:** Demographic characteristics of the sample, Sukhothai, 2019 (N=462)

*Characteristics*	*n*	*%*
**Gender**		
Male	329	71.20
Female	133	28.80
**Age** (years)		
≤40	191	41.30
≥41	271	58.70
**Overtime work**		
Yes	187	40.50
No	275	59.50
**Education level**		
Primary school	288	62.30
Secondary school and higher	174	37.70
**Duration of staying at the worker camp** (years)		
<2	157	34.00
≥2	305	66.00
**Cigarette shop in the camp**		
Yes	342	74.00
No	120	26.00

**Table 3:** Analysis of relationships between population attributes and exposure to secondhand smoke, Sukhothai, 2019 (N=462)

*Factors*	*Total n (%)*	*Exposure to secondhand smoke*		*p*
		*Yes n (%)*	*No n (%)*
**Gender**				
Male	133 (28.8)	83 (62.4)	50 (37.6)	0.520
Female	329 (71.2)	216 (65.7)	113 (34.3)	
**Age** (years)				
≤40	191 (41.3)	143 (74.9)	48 (25.1)	<0.001
≥41	271 (58.7)	156 (57.6)	115 (42.4)	
**Overtime work**				
Yes	187 (40.5)	142 (75.9)	45 (24.1)	<0.001
No	275 (59.5)	157 (57.1)	118 (42.9)	
**Education level**				
Primary school	174 (37.7)	109 (62.6)	65 (37.4)	0.483
Secondary school and higher	288 (62.3)	190 (66.0)	98 (34.0)	
**Duration staying at the worker camp** (years)				
<2	157 (34.0)	91 (58.0)	66 (42.0)	0.031
≥2	305 (66.0)	208 (68.2)	97 (31.8)	
**Cigarette shop in the camp**				
Yes	342 (74.0)	222 (64.9)	120 (35.1)	0.912
No	120 (26.0)	77 (64.2)	43 (35.8)	

**Table 4:** Logistic regression analysis between population attributes and exposure to secondhand smoke, Sukhothai, 2019 (N=462)

*Factors*	*Total n (%)*	*Exposure to secondhand smoke*		*OR (95% CI)*	*p*
		*Yes n (%)*	*No n (%)*		
**Age** ( years )					
≤40	191 (41.3)	143 (74.9)	48 (25.1)	1.932 (1.24–3.01)	0.004
≥41	271 (58.7)	156 (57.6)	115 (42.4)		
**Overtime work**					
Yes	187 (40.5)	142 (75.9)	45 (24.1)	1.717 (1.10–2.66)	0.016
No	275 (59.5)	157 (57.1)	118 (42.9)		

Before adjustment using χ^2^, the significant factors were age, overtime work, and duration staying at the worker camp. After adjustment using logistic regression, the significant factors were age and overtime work.

### Qualitative method

From the general information, 59.1% of the workers joining in the group discussion were males while 40.9% were female, and 30.1% were aged 30–40 years; the mean age was 36 years. Most completed primary school (74.3%), followed by secondary school (17.4%). Only 6.3% were illiterate. The majority were married (78.8%), whereas the rest were single (18.2%) and divorced (2.9%), while 73.6% earned 2501–6000 Tai Baht per month (about US$31 to 1000 THB). Group discussion results were as follows.

#### General context

Sukhothai is famous for growing sugarcane, and many sugar factories are located in the province. Seasonal informal sugarcane harvest workers are coming to the province from December to April each year. Most are from the northeastern and northern regions of Thailand. Most camps were built with galvanized iron sheets in the worker camp, whereas some were constructed with bamboo with the galvanized iron roof for accommodation. The number of workers at each camp was about 50–100. The camps were primarily set in the sugarcane fields or the employer’s house area.

#### Smoking

The number of smokers in the camp increased every year. Some started smoking this year because their smoker friends convinced them. The group opinion illustrated that working in the provinces and staying in the camp for many months might change the nondrinkers and non-smokers to drinkers and smokers because of their friends’ persuasion. Further, the possibility of smoking and drinking of the nondrinkers and non-smokers was higher when they were gathering.

#### Factors leading to exposure to secondhand smoke

##### Chatting and smoking during free time

The workers had free time after work. During this time, women workers prepared the meal while male workers were chatting and smoking in front of the room. After dinner, women workers and children gathered and watched television in the hall in the camp center, whereas male workers continued smoking.

##### Drinking alcohol led to smoking

Drinking alcohol after work at the sugarcane harvest worker camp is very common. The workers contributed money to buy rice whiskey and gathered in circles to drink it from one plastic glass around the circle. The side dish was roasted tamarind seeds. They believed that drinking alcohol relieved the pain and aches from working and helped them to have a good sleep. Moreover, some drinkers shared their cigarettes with their friends, so new smokers were from the drinkers’ group.

##### Cheap cigarettes

Roll-up cigarettes were popular among people with low income and agriculturists in rural areas because of the low price and availability. The workers bought them from the flea markets or the grocery shops in the camp. Some had the belief that smoking roll-up cigarettes were not harmful to health.

##### Exposure to secondhand smoke

The members of the discussion group had different experiences of exposure to secondhand smoke. Some were exposed from their husbands, colleagues, or the teenagers in the sugarcane fields. Morning and evening at the camp area were when they were exposed to secondhand smoke the most because the workers were living together at the camp; the exposure locations were the eating spaces, toilets, quad, bedroom while watching television, and in bed. They were also exposed to secondhand smoke while travelling from the camp to the worksite in the morning and evening and at the harvest area.

Table 5 illustrates that before implementing the measures (by the workers), there was 45.5% SHS exposure in front of the room, followed by the main hall (33.8%) and inside the room (32.3%). After implementing the measures (by the same workers), only 5.8% of SHS exposure occurred in the room, whereas none smoked in the main hall and inside the room.

**Table 5:** Evaluation results of social measures effectiveness, Sukhothai, 2019

*Location of secondhand smoke exposure*	*Before measures implementation (n=68)*		*After measures implementation (n=68)*	
	*n*	*%*	*n*	*%*
1. In the room	22	32.35	0	0
2. In front of the room	31	45.58	2	5.8
3. At the corner in the camp	19	27.94	1	2.9
4. In the toilet	18	26.47	2	5.8
5. Public bathroom	12	17.64	2	5.8
6. Main hall for watching television	23	33.82	0	0
7. Camp entrance	21	30.88	1	2.9
8. Cooking area	10	14.70	0	0
9. At the quad in the center of the camp	13	19.11	0	0
10. At the washing area	8	11.76	2	5.8

#### Results of lessons learned on the social measures

##### Employer

The employer played a crucial role in moving the social measures forward to control and prevent new smokers and protect the non-smokers’ health among the migrant workers at the worker camp. The employer selected and invited the workers, who might be in the same group as the previous year, to work and stay at the camp. For this reason, the relationship between the employer and workers is quite positive. Consequently, the request not to smoke in the camp is heeded and the workers also showed respect to the employer. Furthermore, some families received their pay in advance and returned to work to repay in the following year, so they were considerate and did not refuse what the employer was asking them to do. Besides, many employers offered help to their workers regarding essentials such as rice, consumer goods, water, and electricity to persuade them to consider coming to work with them again in the following year.

##### Leader of the workers

The workers’ leader controlled and monitored the workers’ orders and coordinated with the employer. The leader of workers was selected by voting from all the workers. Therefore, the workers had a close relationship with the leader. He might be a friend, relative, or someone from the same village who worked in the camp. Living together was the opportunity for them to talk and persuade smoking workers to limit the smoking time and not publicly smoke. The leader customarily cooked, travelled to the field, and ate together with the workers, so they had adequate time to talk and warn each other.

##### Housewives

Some of the housewives’ prominent roles were cooking, taking care of the child, and working in the field with the husband. They would ask the husband to smoke outside the camp because they were aware of the impacts of smoke and their children’s health. For this reason, the housewives were the influential people who controlled smoking in the worker camp effectively.

##### Public health officers

The key roles were to take care of the health of the workers in the camp and educate them about SHS exposure via various channels, such as pamphlets, posters, no-smoking stickers, individual training about health education, and group feedback to the community to inform them of the smoking and SHS exposure situation, as well as the impacts on the workers’ health. As a result, the workers started to show concern about the harm of secondhand smoke. In this research, the public health officer enforced the social measures by giving examples from experience in the care of patients with smoking problems to create awareness and motivation to reduce the frequency and quit smoking.

## DISCUSSION

The results showed that the different social measures include self-awareness, social norms, the arrangement of an environment facilitating the new norms, empowering women to stop the husbands who smoked in the camp and the fields, and reducing free time to distract the workers from smoking reduced SHS exposure. Moreover, the measures also included not placing cigarettes in visible places that made it easy to smoke in the neighboring camps, no smoking in the main hall where the women and children watched television and minimizing drinking alcohol in the camp to reduce smoking while drinking, all helped reduce SHS exposure. After one month of implementing the measures, the evaluation results illustrated that the experimental group was less exposed to secondhand smoke. The relatives and family members smoked less and were able to warn smokers. Moreover, the employer cooperated in giving warnings. Self-awareness has been found a significant attribute to the cession of smoking, this helps to reduce SHS exposure^15^. Besides, a recent study conducted in Bangladesh found that practicing social norms helped reduce SHS exposure among children^16^. The result also was in line with another study in Canada among young adults^17^. Our study showed woman empowerment as a significant factor in line with other studies conducted in South Asia^16,18^. Smoking in a visible place increases a non-smoker’s chances to start smoking and increasing SHS, which was also found in a study conducted in England^19^. Besides alcohol drinking increases smoking as was also found by previous research^20^. In addition, our research also found long working hours result in working stress which is related to smoking, also in line with a study from Finland^21^.

The social measures to protect the health of migrant workers and families from SHS exposure were determined based on the perspectives of the relevant people and the root of the problems. The measures were developed from informal retrieval and the participation and support from the relevant sectors, including the employers, officers, and shop owners, who expressed their opinions, made decisions and took part in the operation via the public hearing until the workers’ community accepted and processed the solutions by themselves. However, some aspects were unable to be processed, such as academic information for which the assistance of the government sector was required. It is evident that social measures needed support from the public health officers to operate, entrepreneurs’ decision-making to control and monitor, and the grocery shops to set the market mechanism and implement the social measures.

### Strengths and limitations

Social measures are a significant strength of the present research, used also in other settings to minimize the SHS exposure. Despite this strength, we used a cross-sectional study in one of the phases, which only gives a particular time picture. Besides, in the group discussions, the sample size was small, which may affect the outcome, and one month is a very short duration to evaluate the effect of social change measures. The study also neglected populations in Thailand that moved around the country for employment.

## CONCLUSIONS

Our research is one of the first of this kind of study focused on the sugarcane migrant workers’ exposure to secondhand smoke. The results showed that social measures help smokers to reduce secondhand smoke exposure to their close ones. For the sustainability of any social measure, a long-term evaluation is recommended to get proper outcomes.

## Data Availability

The data supporting this research are available from the authors on reasonable request.
